# Allergies, *Helicobacter pylori* and the continental enigmas

**DOI:** 10.3389/fmicb.2015.00578

**Published:** 2015-06-09

**Authors:** Ramakrishnan Sitaraman

**Affiliations:** Department of Biotechnology, TERI University, New Delhi, India

**Keywords:** *Helicobacter pylori*, allergy, Asian enigma, African enigma, immunomodulation, hygiene hypothesis, microbiota, ecology and evolution

## Abstract

*Helicobacter pylori*, a gastric pathogen, is known to be associated with gastric and duodenal ulcers, and is also a strong risk factor for the development of gastric cancer and lymphoma of the mucosal-associated lymphoid tissue. Ordinarily, this should make a strong case for its eradication at par with any other infectious disease. However, the unique biology of *H. pylori* and the complexity of its interactions with humans, its only known natural host, do not permit the recommendation of unambiguous preventive and therapeutic measures. Moreover, this organism has co-evolved with humans as a practically universal member of the natural gastric microbiota over at least 100,000 years. *H. pylori* persists for a lifetime in mostly asymptomatic hosts, and causes clinical disease only in a minority of infections. Therefore, its potential contribution to the maintenance of human immune homeostasis, as is the case with the better-studied members of the intestinal microbiota, is certainly worthy of serious investigation. In this paper, we summarize some interesting and often anecdotal data drawn from recent studies, and examine their significance in the context of the hygiene hypothesis. We also examine whether the lower incidence of gastric cancer over large parts of the world in spite of a high prevalence of infection (the Asian and African enigmas) may be re-interpreted in terms of the hygiene hypothesis. Finally, it is suggested that an evolutionary-ecological approach to the study of *H. pylori* infection may help in the formulation of strategies for the management of this infection. This may well be an infectious disease wherein medical interventions may have to be personalized to ensure optimal outcomes.

## Introduction

Ever since its identification in 1983 as the causative agent of gastric ulcers by Barry Marshall and Robin Warren, *Helicobacter pylori* has been recognized as an unusual component of the human gut microbiota. While the intestinal microbiota, more numerous and varied, have attracted much attention from researchers, studies of the stomach microbiota are much fewer and far between. Therefore, because of fortuitous recovery in pure culture and the recognition of its pathogenic potential, *H. pylori* is the best-studied member of the gastric microbiota to date. Though its pathogenic aspect has been investigated in much depth, examining the role of *H. pylori* as a constituent of the resident stomach microbiota can potentially illuminate the evolutionary value of this ancient association.

Typically, for a chronic pathogen, it is expected that natural selection will tend to favor less virulent strains. Indeed, a majority of *H. pylori*-infected people are asymptomatic carriers. However, it also raises the evolutionarily tenable possibility that chronic infection by a less virulent pathogen might also confer some adaptive benefits on the host. The “hygiene hypothesis” states that exposure to infectious agents, especially early in life, “trains” the host immune system resulting in a decreased tendency to subsequently develop allergies. In this article, we examine the so-called “enigmas” associated with *H. pylori*, i.e., the lower incidence of *H. pylori*-associated disease (gastric cancer, gastric, and duodenal ulcers) in spite of a high incidence of *H. pylori* infection in certain populations of the world, in the context of the hygiene hypothesis.

## The Continental Enigmas Vis-à-vis the Hygiene Hypothesis

We introduce the term “continental enigma” to refer to the counterintuitive observations from a pathogenesis-based viewpoint that high infection rates with *H. pylori* over extensive areas of Asia, Africa, and parts of Central/South America are not accompanied by a correspondingly higher incidence of clinical symptoms ranging from gastric and duodenal ulcers to gastric cancer (see Holcombe, 1992; Miwa et al., 2002; Singh and Ghoshal, 2006; Misra et al., 2014). Instead, most infected individuals exhibit only asymptomatic gastritis. The ubiquity of *H. pylori* infection (∼50% of the world’s population) and the seeming lack of a corresponding disease burden in terms of morbidity and mortality indicates that this bacterium is probably not a conventional pathogen predictably causing disease. Rather, the clinical outcome is found to be dependent on the infecting bacterial strain, the host genetic background as well as environmental factors such as dietary habits and smoking.

The term “hygiene hypothesis” has its origins in the observation by David Strachan in the UK that children with larger numbers of older siblings tended to be less likely to develop susceptibility to hay fever (allergic rhinitis, Strachan et al., 1996). This was considered as the effect of repeated cross-infections from older siblings during childhood, i.e., childhood infections somehow decreased the probability of developing allergies later in life. With time, the hygiene hypothesis has been extensively reformulated to cover not only allergies but also atopy and autoimmune disorders. In its current form, the hygiene hypothesis states that exposure to various organisms—commensals, symbionts, mutualists and pathogens, both unicellular (microbes) and multicellular (parasitic helminths)—during early childhood lowers the risk of developing immune disorders later in life. However, the question of mechanism and its dependence on a particular member(s) of the resident and transient microbiota in given contexts is yet to be addressed in sufficient detail. Ideally, we would like to know whether a specific microbiota-derived component(s) acts as an immunomodulator, and the mechanism of its action. Importantly, this does not preclude redundancy among microbiota members and components in their ability to act as immunomodulators.

### The Antiquity of the Human-*H. pylori* Association

Research over the last 15 years has made it amply clear that the association between *H. pylori* and its only known host—humans—is considerably ancient. The bacterium has been detected in a pre-Columbian Mexican mummy (Castillo-Rojas et al., 2008), and the clustering of *H. pylori* strain haplotypes closely aligns with known human migrations out of Africa and subsequent settlement in other continents (Linz et al., 2007; Moodley et al., 2012). The clustering of *H. pylori* strain haplotypes may even be used as surrogate markers to infer historical human migration patterns (Falush et al., 2003). *H. pylori* infection prevalence may also serve to clarify or refine inferences of both historical migration and isolation, as was demonstrated in a recent study on the North Sulawesi island of the Indonesian archipelago (Miftahussurur et al., 2014). Among other studies of contemporary humans, the study of relatively isolated native South American tribal villages found a high prevalence of *H. pylori* infection as measured by antibodies to CagA, VacA, urease A (30 kDa), and flagella (Robinson et al., 2002). In that study, even the tribes that had remained fairly isolated and had experienced first contact with the external world very recently at the time of sample collection, exhibited a high prevalence of *H. pylori* infection amounting to 100% in some instances. Notably, seropositivity to *H. pylori* antigens was detected at 100% prevalence among the Ewarhoyana (first contact -1970, 0.1 years before sample collection), 98% among the Parakana C (first contact -1984, 0.2 years before sample collection), and 87% amongh the Parakana Novo (first contact -1977, 0.2 years before sample collection). These observations further buttress the thesis of an ancient association of *H. pylori* with humans, its only known natural host.

### The Variable Effects of an Ancient Association

While the continental enigmas are indicative of some trends over large (continent-scale) areas, a closer look indicates significant variation within the populations involved. It must also be pointed out that human populations, even when geographically close together, are not uniform in terms of symptoms, probably due to a combination of host genetics and environmental influences. For example, a direct comparison of Indian urban (Bengali) and tribal populations (Santhal and Oraon) living in close proximity revealed similar *H. pylori* prevalence, but also found that the tribal population was less likely to manifest symptoms of duodenal ulceration upon infection (Saha et al., 2009). Earlier analysis of the same tribal populations indicated high *H. pylori* prevalence, and a similar occurrence rate of virulence-associated genes (*cagA* and *vacA*), but none of the subjects had any peptic ulcer diseases (Datta et al., 2003).

Epidemiological information from Malaysia presents a strong case for exercising caution when predicting the effects of *H. pylori* infection, beneficial or not, on the host. The Malay population consistently exhibits a lower prevalence of *H. pylori* compared to the ethnic Indian and Chinese populations. Interestingly, while practically all peptic ulcer patients from all three groups are infected with *H. pylori*, a lower percentage of infected Indians presented with peptic ulcer disease compared to Malays (Goh and Parasakthi, 2001). The strong influence of ethnicity on the outcome of *H. pylori* infection has been termed the “racial cohort phenomenon” (Goh, 1999). Asymptomatic and widespread carriage among hosts of Indian ancestry could also be indicative of an unknown adaptive benefit to the host.

### *H. pylori* and Allergies

Studies conducted in the recent past have indicated that the decreasing incidence of *H. pylori* in the developed world is paralleled by an increase in the incidence of childhood allergies and autoimmune diseases. Analysis of data from the third National Health and Nutrition Examination Survey (NHANES) conducted during (1988–1994) in the U.S.A., compiling data from 7663 adults first revealed this trend (National Center for Health Statistics, 1994). An inverse correlation was observed between prior acquisition of *H. pylori* and the possibility of being diagnosed with asthma or allergy. It was also found that infection with *cagA*^+^ strains of *H. pylori* was associated with a reduced risk of childhood-onset (< = 15 years) asthma (Chen and Blaser, 2007). Subsequently, data from the fourth NHANES survey (1999-2000) (National Center for Health Statistics, 2005) covering 7412 individuals (out of 8969 enrolled participants), that included not only adults but children and teenagers, indicated that acquiring *H. pylori* infection earlier in life was correlated with a lower occurrence of allergies and asthma (Chen and Blaser, 2008). A more recent study of allergic rhinitis among 97 healthy Japanese volunteers, though generally in agreement with the NHANES-based findings regarding childhood allergies, also exhibited a lack of correlation between *H. pylori* infection and allergic conditions in subjects 50 years or older. This indicates that other factors such as the social environment might also be involved in the final outcome (Imamura et al., 2010). The most recent meta-analysis of available studies found “weak evidence” for an inverse association between *H. pylori* infection in the adult and pediatric populations studied (Wang et al., 2013). Thus, it would not be unreasonable to expect that, as an ancient member of the stomach microbiota and a chronic perister, *H. pylori* could be one of the bacteria contributing to the “hygiene effect” on the development of allergies.

Recently, significant progress has been made in identifying a molecular mechanism underlying the protective effect of *H. pylori* infection, mediated via the generation of tolerogenic dendritic cells (DCs), using a mouse model of ovalbumin sensitization and challenge (Oertli et al., 2012). In preliminary experiments, *in vitro* co-culture with *H. pylori* was found to inhibit the maturation of murine bone-marrow derived DCs on exposure to *E. coli*-derived acellular lipopolysaccharide (LPS). The murine DCs co-cultured with *H. pylori*, but not naïve murine DCs, were found to be capable of converting murine T cells to T regulatory cells (Tregs) *in vitro*. Ovalbumin-loaded DCs were capable of inducing tolerance during ovalbumin challenge in live mice, provided they were also exposed to *H. pylori* before transfer. Mice infected neonatally, rather than as adults, upon infection with a virulent *cagA*^+^ *H. pylori* isolate, were found to be more densely colonized and harbored tolerogenic DCs. IL-18 secreted by tolerogenic DCs was found to be crucial in mediating the conversion of T cells to Tregs. The “hygiene effect” of *H. pylori* infection is therefore predicated on its ability to persist in human host, often over a lifetime.

From the viewpoint of epidemiology, data on the simultaneous prevalence of *H. pylori* and allergies is scarce outside the developed world. Whether the purported inverse relationship between *H. pylori* prevalence and risk of allergies holds true the world over therefore remains uncertain. A particularly interesting example is again the low prevalence of *H. pylori* infection in the Malay population, within an overall context of poor household hygiene and limited antibiotic use. In a recent review, it was found that a lower prevalence of *H. pylori* infection was indeed associated with a lower disease burden (Lee et al., 2013). However, the very low prevalence of both *H. pylori* and childhood asthma among Malaysian children suggests that *H. pylori* infection in high-prevalence populations may be a surrogate marker of poor household hygiene, rather than being independently protective against asthma (Chen and Blaser, 2009; Raj et al., 2009). Or, as stated earlier, the mechanism(s) of immunomodulation by *H. pylori* are not unique, and other infectious agents might be able to achieve a similar outcome viz., reducing the likelihood of developing allergies.

Finally, the “continental enigmas” provide an indirect indication that the final outcome of *H. pylori* infection could be very different based not only on the bacterial strain and host genetics, but also on the stage of life at which the infection is first acquired. A plausible model would be that acquisition of *H. pylori* during early childhood has a protective effect with respect to the development of allergies later in life. By contrast, if *H. pylori* infection during childhood were to be prevented or disrupted with sanitary measures and medication, as in developed nations, infection later in life is more likely to result in pathogenesis. In such a view, the so-called continental enigmas arise as a consequence of to the historical accident of retrospectively applying a pathogenesis-oriented framework derived from late-acquired *H. pylori* infections in the developed world, to other regions where infections early in life are more normative.

## Some Additional Considerations

As pointed out in earlier sections, *H. pylori* is but one, albeit important, member of the human stomach microbiota. There is also significant variability associated with its presence in different human populations across both geography and ethnicity. Here, we consider some interesting and/or speculative aspects of the human-*H. pylori* association within an ecological-evolutionary framework.

### *H. pylori* Interactions with the Stomach Microbiota

Recently, an interesting study examined the effect of pre-existing stomach microbiota on the consequences of subsequent *H. pylori* infection in a mouse model (Rolig et al., 2013). From an ecological perspective, this study evaluated the resilience of the stomach microbiota to perturbations by a potential pathogen. It demonstrated that interactions between the pre-existing ecosystem and the new entrant potentially impact the environmental niche viz. inflammatory responses in the murine stomach. Initially, it was observed that the same strain of mice (C57BL/6N) procured from two different vendors differed in initial compositions of the stomach microbiota and also exhibited different degrees of inflammation upon experimental infection with the mouse-adapted *H. pylori* strain SS1. CD4^+^ T cell infiltration into the mouse stomach was found to be consistently lower when experimental *H. pylori* infection was preceded by antibiotic treatment of the mice. While the overall composition of the microbiota did not change significantly post-infection, there was some effect on individual members of the microbiota—notably, an increase in cluster IV and XIVa *Clostridium* spp. There was also a lower degree of inflammation and a lack of the T-helper type 1 (Th1) cytokine, interferon-γ. As the authors point out, this study does not rule out perturbations of microbiota at other sites along the gastrointestinal tract, or conversely, their influence on the incoming infection. And, as the study was carried out over 4 weeks, the long-term effects as well as the persistence of the effects observed during this period remain unknown.

### Evolutionary History—Ancient and Recent

The importance of evolutionary history to the clinical outcome of *H. pylori* infection was highlighted in a study of geographically separated Colombian communities that experienced different levels of European contact and genetic admixture (Kodaman et al., 2014). This study indicated that introduction of a new haplotypes of *H. pylori* into formerly isolated populations during genetic admixture significantly disturbed the co-evolutionary equilibrium between a chronic infectious agent and its host. Thus, greater European ancestry among the mestizo communities in the mountains was associated with the replacement of Amerindian *H. pylori* strains with European strains. By contrast, costal communities of African ancestry were more commonly infected with ancestral African-type strains. All the communities studied exhibited a high prevalence of *H. pylori* infection (∼90%), but vastly different incidence rates of gastric cancer (150 per 10^5^ in the highlands versus 6 per 10^5^ on the coast). Incidentally, this gives some insight into the continental enigmas, suggesting that co-evolution of particular bacterial and host genotypes over extended periods of time may have moderated adverse outcomes for the host. This equilibrium could then be disturbed as a consequence of migrations, invasions or racial admixture.

### Is the Greater Incidence of Allergy Partly Attributable to Greater Human Variability?

Since 1750, when the estimated world population was around 770 million, the human population stands at seven billion today, and increase of nearly an order of magnitude. From 1950 onweards, the human population has grown at the annual rate of 1.8 per cent (Hirschman, 2005). Sanitation limits pathogen exposure and its benefits in terms of reduction of morbidity and mortality are undeniable. Antimicrobial agents cut short what might be called the “natural course” of infection. Vaccination, especially with recombinant or purified immunogenic components of pathogens, also pre-emptively short-circuits infections. It therefore stands to reason that this minimization of strong selection forces exerted by life-threatening pathogen exposure and infection coincidentally alleviated a genetic bottleneck effect. This can, in turn, potentially allow greater genetic diversity among humans. In other words, the pre-industrial revolution populations could have been genetically less diverse. It is conceivable that some of these variations could be those that predispose to the development of allergies. Indirect support for this idea comes from observations of the selective inactivation of the sialic acid-recognizing Ig-like lectin (*SIGLEC*) genes that encode a family of transmembrane receptors, within the hominin lineage (Wang et al., 2012). These receptors are predominantly expressed on innate immune cells, and modulate responses to pathogens. Within the hominin lineage, *SIGLEC- 13* and *-17* have been inactivated, and the authors suggest that this may be due to two, mutually non-exclusive reasons. Lethal infections caused by pathogen engagement of these Siglecs could have resulted in strong negative selection. The engagement of these Siglecs could also have resulted in toxic inflammatory effects in ancestral hominins. Therefore, the observed effect of improved hygiene may be attributable to the greater diversity of immune responses that are now prevalent in the population. Higher chances of long-term survival for larger numbers of people, and the mitigation of strong negative selection imposed by pathogens could therefore manifest as an increase in the prevalence of allergies and immune disorders. This is admittedly speculative, but has not been proposed or considered before, to the best of my knowledge.

## Concluding Remarks

As is evident from the foregoing account, there is no unequivocal answer to the clinical question as to whether *H. pylori* is a human pathogen that warrants elimination on a global scale. Definitively attributing clinical outcomes to a combination of *H. pylori* strains, their virulence factors, the host genetic background and environmental factors is a daunting task. Even if such information were forthcoming and large-scale trends discernable, it would imply that intervention in individual cases will have to be personalized, ranging from passive non-interference to aggressive treatment and periodical monitoring for re-infection after successful therapy. On the other hand, the existence of *H. pylori* in human stomachs for millennia and its potential to persist in the human stomach over a lifetime provides us a unique opportunity to identify microbial (bacterial) and host factors that lead to a decrease in the likelihood of developing atopy in general, and allergies in particular. When our knowledge of these factors is combined with mechanistic information, it may well be found that *H. pylori* is not unique in its immunomodulating capabilities relative to other commensals or pathogens. However, our detailed knowledge of the biology of *H. pylori* and the process of pathogenesis within the context of the continental enigmas has the potential to serve as a useful model system to delineate multiple aspects of immunomodulation over a lifetime by key members of the microbiota. It offers us a unique opportunity to analyze the evolutionary and ecological adjustments and trade-offs underlying the establishment and maintenance of the host-microbiota association, providing valuable information with predictive power in the quest for a better quality of life.

### Conflict of Interest Statement

The author declares that the research was conducted in the absence of any commercial or financial relationships that could be construed as a potential conflict of interest.
